# Process makes perfect: mapping science team behavior across phases

**DOI:** 10.3389/fpsyg.2026.1729978

**Published:** 2026-03-25

**Authors:** Angie N. Benda, William S. Kramer, Hanna M. Johnston, Kelsey Ciagala, Danielle J. Crawford, Kayla N. Lacey

**Affiliations:** Department of Psychology, University of Nebraska at Omaha, Omaha, NE, United States

**Keywords:** collaboration, science of team science, science teams, team processes, translational research

## Abstract

Modern technological and methodological advancements in science require experts to collaborate to answer more complex questions. Science teams are uniquely equipped with expertise to explore novel approaches to answer these questions. Prior research has advanced the understanding of science teams through stage-based models, but a gap remains in integrating these models with process models. To address this gap, we draw on two foundational frameworks—the four-phased transdisciplinary team development model and the temporally grounded framework of team processes—to develop an integrated conceptual framework of science team behavior. We synthesize these two frameworks using a conceptual integration approach to map team processes with the focal activities that characterize the four phases of science teams. This integration highlights the criticality of transition processes in shaping coordination and the development of shared knowledge of team progress. The resulting framework offers insight into the dynamic nature of science teams and their processes, identifying what is most salient in each stage and what shifts as teams adapt. In doing so, we advance team science by elaborating on challenges that may be encountered and provide testable propositions to guide future empirical research and further support science teams.

## 1 Introduction

Science teams have become a staple for producing high-impact research that addresses complex questions requiring multiple forms of expertise. Most recently, COVID-19 was a high-profile, public-facing illustration of the power of science teams to rapidly integrate knowledge under conditions that were time-sensitive and highly consequential (Natale et al., 2020). Interest in understanding science teams is relatively recent and expected to continue to grow, particularly following the report *The Science and Practice of Team Science* from the National Academies of Sciences, Engineering, and Medicine (2025), which details guidelines for developing effective scientific collaborations.

Science teams often span organizations and disciplines, bringing together diverse knowledge to solve real-world problems and generate collective understanding (Fiore, 2008; Martinez Tyson et al., 2025; Stokols et al., 2008). Unlike traditional work teams that tend to be stable and output-focused, science teams operate in dynamic environments where members’ influence may shift across research phases (Börner et al., 2010). They also face evolving research goals, changing institutional constraints, and fluid team membership as collaborators join or leave (Cummings and Kiesler, 2007). Such complexity calls for a framework that can account not only for what science teams do, but also *when* and *how* key processes unfold.

Research on science teams often emphasizes broad developmental stages or contextual influences, whereas traditional team process models focus on the behavioral dynamics that transpire within shorter task cycles. As a result, few frameworks integrate when science teams encounter key challenges with how they are navigated. To address this gap, we propose an integrative framework that aligns Hall et al.’s (2012) four-phase model of transdisciplinary team development with Marks et al.’s (2001) temporally based team process model. Since Hall et al.’s (2012) phases describe when science teams undergo major developmental shifts, and Marks et al.’s processes describe how teams plan, act, and relate within those periods, integrating them allows us to locate specific teamwork processes within the broader lifecycle of science teams. This integration addresses calls in the Science of Team Science (SciTS) literature for models that explicitly link when teams encounter key challenges with how they navigate them. This integration connects the macro-level evolution of scientific collaboration with the micro-level processes that sustain team functioning across time, offering a comprehensive view of the cyclical and recursive nature of science teams.

Despite rich work on team processes and science teams, there is still no integrative framework that links these literatures by connecting temporally grounded process models with the developmental phases that characterize scientific collaboration. Existing approaches, such as input-process-output (I-P-O) and input-mediator-output-input (I-M-O-I) models, acknowledge that teams unfold over time, but they typically treat time as a backdrop rather than specifying how particular behavioral mechanisms map onto distinct stages of problem formulation, implementation, and translation. Our framework addresses this gap by offering a dynamic, phase-based process model that locates concrete behavioral interactions within each stage of a science team’s lifecycle, providing a clearer path for anticipating coordination challenges and selecting appropriate strategies as teams progress toward their goals. Accordingly, the objectives of this paper are twofold: (1) to integrate a macro phase-based model with a temporally grounded team process model, specifically stating the most salient processes at each phase; (2) to demonstrate our framework’s application across different types of science teams. We contribute to current bodies of work by developing phase-specific propositions and offering testable pathways for advancing theory and practice.

## 2 Defining science teams and contextual factors

As our framework focuses on how team processes unfold across phases of scientific collaboration, it is important to first clarify what distinguishes science teams from others. A team is generally defined as two or more individuals who interact interdependently and adaptively, each fulfilling distinct roles in pursuit of a common goal (Kozlowski and Ilgen, 2006; Salas et al., 1992). While this broad definition provides a useful foundation, science teams represent a specific and increasingly prevalent form of collaboration that differs from traditional teams in key ways.

Science teams vary substantially in structure and composition, so we treat them as a broad category rather than a single team type. Disciplinary breadth spans a continuum from unidisciplinary teams, where members share a common scientific background, to multidisciplinary and interdisciplinary teams, and ultimately to transdisciplinary teams that integrate theories and methods across fields to generate new frameworks and approaches. Geographic dispersion, role differentiation, and project duration also vary across this continuum and across science teams more generally. The framework developed here is intended to apply across this range while clarifying how core processes become more or less salient as teams differ in disciplinary breadth and related coordination demands.

Across science teams, three recurring features often shape coordination demands: (1) functional diversity (Fiore, 2008; Salazar et al., 2012; Stokols et al., 2008), (2) temporal complexity (Cummings and Kiesler, 2007), and (3) adaptive goals (Börner et al., 2010; Hall et al., 2012). Unlike project or management teams that pursue a well-defined goal within a single organizational context, science teams operate in a dynamic landscape where goals evolve as new insights emerge, and team composition shifts. Their success depends not only on achieving outcomes but also on integrating diverse perspectives, managing boundary-spanning relationships, and sustaining collaboration over extended periods (Hall et al., 2018).

Science teams thus represent a distinct class of teams characterized by functional diversity, adaptive goals, and temporal complexity. These attributes make them ideal for a process-oriented, temporal framework that can account for the dynamic behaviors and the developmental stages that shape collaboration over time. Understanding these unique features also clarifies why models originally developed for co-located, task-focused teams (e.g., Marks et al., 2001) may not fully capture the evolving nature of scientific work. By situating science teams within this broader theoretical landscape, we set the stage for integrating process-based and phase-based models to describe their operation more precisely. It is also important to recognize the influence of contextual and compositional factors and how science teams interact. These factors can moderate science teams, altering their affect, behavior, and cognition. We briefly discuss the impact of knowledge diversity, project length, and team member location, and what impact they would have on science teams.

### 2.1 Knowledge diversity in science teams

A central compositional feature of science teams is the breadth of knowledge and functional roles within them. Functional diversity refers to the premise that each team member contributes varying degrees of information, perspective, and expertise to the team. Science teams exist on a continuum spanning from unidisciplinary to transdisciplinary makeup, reflecting varying degrees of shared knowledge and unique, non-overlapping variation within the team (Chen et al., 2025; Specht and Crowston, 2022).

Functional diversity can moderate team processes and outcomes. Increased functional diversity may create more task conflict (Weingart et al., 2000) as multiple approaches are advocated for, and deciding on a course of action may be challenging. Communication barriers may also arise in teams with greater functional diversity because of the unique terminology used across disciplines. However, increased functional diversity also generates more novel and complex approaches (Abiew et al., 2022). For science teams specifically, as research questions grow more sophisticated, increased functional diversity can allow teams to answer questions from different viewpoints. All in all, functional diversity is context-dependent and can both complicate and enrich the collaborative process that is critical for science teams.

### 2.2 Project length

Science teams also vary substantially in project duration, ranging from short-term collaborations to initiatives spanning decades. Project length affects team composition and processes. In long-term project teams, membership is likely to change over time, making them more dynamic, and teams may experience unstable membership. In these cases, dynamic membership can alter teams’ shared processes [e.g., shared mental model (SMM), coordination efforts], which may need to be re-evaluated and updated as team membership shifts (Bell and Fisher, 2012; Kozlowski, 2015; Tannenbaum et al., 2012). For example, when new members are brought in, the team may experience recursion to earlier planning stages as they onboard new members and incorporate additional perspectives that can be integrated into the current problem set. In contrast, short-term science teams may remain together as a unit for the whole project duration. In these cases, teams may more readily build trust, shared understanding, and psychological safety, particularly when it is known from the beginning that their time is limited (Edmondson, 1999). Regardless of the project duration, the anticipated length of the project impacts the processes that unfold across a team’s lifecycle, highlighting the role of temporality.

### 2.3 Science team location

Finally, team member location impacts the processes science teams engage in at each stage. For teams that are co-located and have consistent in-person meetings, work may progress more quickly and include more informal touchpoints than teams that are not co-located (Hall et al., 2018). Given that science teams are often composed of highly specialized individuals with diverse knowledge and skills, it is likely that their members engage in dispersed work, as recruitment can occur without the limitation of physical proximity (Tzabbar and Baburaj, 2019). Teams that work in a dispersed manner may not only differ in their physical location but also in time zones and technological contexts (Kirkman and Mathieu, 2005).

Teams operating in dispersed environments must therefore be more intentional about their processes. Establishing communication norms, such as determining the tools that will be used for communication and when meetings will occur, can largely impact the success of the team (Hoegl and Gemuenden, 2001; Marlow et al., 2017; Mors and Waguespack, 2021). Swift trust early in a performance cycle, when the team is first gathering, can help mitigate the consequences of not being in the same place by enabling members to assume others are working on their assigned tasks (Crisp and Jarvenpaa, 2013).

The location of team members becomes increasingly important and must be intentionally managed to sustain productivity. When dispersion reduces both physical and perceived social presence within the team, establishing clear guidelines for when and how interactions should occur, along with developing group norms, helps to foster shared awareness and cohesion (Benda et al., 2023). Ultimately, for science teams, the additional challenge of working in a dispersed manner requires greater effort and structured processes that aid knowledge integration.

Science teams represent a distinct form of collaboration designed to address complex research questions. These teams vary widely in their composition, ranging from relatively homogeneous groups with shared knowledge and roles to highly diverse teams with multiple areas of expertise and training. Contextual factors—including project length and team member location—further influence how science teams develop shared understanding, collaborate, and adapt over time.

## 3 Four-phase model of transdisciplinary team development

To establish the phase-based foundation of our integrative framework, we first present the four-phase model of science team development proposed by Hall et al. (2012). We begin with this model because it was developed in the context of transdisciplinary science teams, which represent the most complex scientific collaborations. The model outlines the stages that transdisciplinary teams move through, beginning with the initial team formation and development and concluding with the translation of research outcomes. Within the broader phases, challenges surrounding coordination and collaboration are also highlighted. Although the Hall et al. (2012) model was developed in the context of transdisciplinary teams, we draw on it here because it offers a clear sequence of phases that capture the developmental patterns present in other compositions of science teams.

Other science team models that could have been selected for our framework include earlier work from, Stokols et al. (2008) and later reviews from Hall et al. (2018). While these frameworks provide context surrounding important considerations for shaping science teams, they offer limited specification of the phases that science teams engage in. Therefore, these limitations motivate the use of the four-phase model as our foundation because it allows for a stronger understanding of what teams are doing at different points in the collaboration process, from team formation through research execution, and the communication of results.

### 3.1 Development phase

The first phase is Development, during which the group begins to form. Transdisciplinary teams may start from existing interests or advisory groups focused on key scientific problems. As initial members ideate about the team’s mission, they build shared awareness and establish psychological safety (Edmondson and Roloff, 2008) so that, as the problem evolves, members feel comfortable voicing their views. Clarifying individual goals early helps create a shared goal and awareness of the expertise present and the outcomes team members want to achieve.

### 3.2 Conceptualization phase

After creating a shared mission and goal, teams begin to build the research plan, developing novel research questions and integrating methods that combine best practices across fields and roles. During this phase, as the research questions take shape, the team may recognize the need to draw on additional expertise and add members to address gaps. Given the scope of Hall et al. (2012), which focused on transdisciplinary teams, disciplinary differences introduce multiple ways of discussing the plan, so this phase emphasizes the creation of a shared language for the research plan. With varied expertise, the team begins to develop a transactive memory system (TMS) that identifies who knows what, and this is updated as the research progresses (Lewis and Herndon, 2011). Once the team believes the integrated research questions and methods are ready, the team moves to Implementation.

### 3.3 Implementation phase

The plan and research questions developed in the Conceptualization phase are enacted during Implementation. Specifically, transdisciplinary teams, composed of applied and academic members, execute the plan to answer the agreed-upon questions and adapt it as needed. They may experience task or relationship conflict and must work through the challenges that arise. Transdisciplinary teams often have minimal overlap in expertise, so each member’s contributions are critical. Persistent or unresolved conflict can stall progress toward the mission and goals. As the research unfolds, the team also updates its SMM and TMS and builds collective knowledge through team learning. The goal in this phase is to generate initial findings that address the questions developed at team formation.

### 3.4 Translation phase

The Translation phase is the final phase in this model of team development. At this point, the team has research findings that were gathered and refined during the Implementation phase. They then develop solutions that can be applied to the guiding mission and questions. Additionally, the team establishes new goals that address how the research will be translated. During this work, the team may shift in composition to bring in new experts who bridge science to real-world applications, speak to the importance of the application, and champion changes to the earlier specified problem or stakeholder group. Much like the Conceptualization phase, the team creates a translation plan, establishes the steps they will take to apply findings, and defines goals to measure success. Some teams, especially those only motivated by basic research questions, may not fully reach Translation by applying their findings, but through publishing their work, it is possible that it creates opportunities for research to be translated by others through the formation of new teams and performance cycles.

Hall et al. (2012) provide a roadmap for transdisciplinary teams, showing the cyclical nature from forming a group to sharing results. An important note is that teams may have to move back to an earlier phase before progressing. For example, during the conceptualization phase, if additional team members are added to support the research goals, they need to be brought into the shared understanding as it exists, which may be updated with the new insights they provide. As teams move between phases, they adapt prior work to reflect the actions and behaviors required in the next phase. Cycling between stages and moving back to an earlier stage is common, especially with complex tasks that require collectively established knowledge, as compared to intradisciplinary work.

## 4 Team process model

To understand how team behaviors unfold across time, we first turn to the temporally based team process model developed by Marks et al. (2001). This framework describes how teams move through recurring cycles of planning, action, and reflection, emphasizing that team performance is inherently dynamic and influenced by time-related factors. Building on the I-P-O tradition (Ilgen et al., 2005), the model conceptualizes teamwork as a series of episodic performance cycles in which the output of one phase becomes the input for the next. In essence, what occurs early in a team’s functioning shapes everything that follows. The model distinguishes between transition, action, and interpersonal phases. This temporal structure provides a useful foundation for understanding the evolving behaviors of science teams, whose work similarly unfolds through iterative and interdependent stages of collaboration.

Similar to science team models, several organizational team models exist, including Tuckman’s staged model (1965), I-M-O-I models (Ilgen et al., 2005; Mathieu et al., 2008), and multilevel models of team effectiveness (Mathieu et al., 2017b). While these frameworks acknowledge that team processes occur over time, they either conceptualize development through broad, generalized phases (Tuckman’s), or situate team dynamics across multiple levels within a system (Mathieu et al., 2017b), offering limited insight into the specific behavioral processes that occur within each phase at the team level. Marks et al. (2001) explicitly detail what is occurring in transition, action, and interpersonal processes as a team engages in performance cycles.

### 4.1 Transition phase processes

Transition phases occur before and between action phases, providing teams with opportunities to plan for future goals and evaluate prior performance episodes. Within each transition phase, three core processes take place: (1) mission analysis, (2) goal specification, and (3) strategy formulation and planning. Together, these processes orient the team toward a shared understanding of the task, define the steps required for success, and create the roadmap that guides action.

Mission analysis marks the first step in a transition phase. Here, teams interpret the task, assess what needs to be achieved, and take inventory of available resources and constraints. This reflection helps the team identify who possesses relevant knowledge and how members’ expertise aligns with the collective mission (Fiore et al., 2001). This process is especially critical in science teams, where evolving goals and membership require periodic reassessment of resources, expertise, and alignment with the broader research mission.

Goal specification follows as teams translate the overarching mission into specific, actionable objectives. This process involves identifying intermediate goals, setting timelines, and clarifying what success looks like for each stage. Formal tools such as team charters can help codify these objectives, promoting commitment and accountability (Rapp, 2025). Clearly defined goals enhance coordination and performance (Mathieu et al., 2008) and, in the context of science teams, help maintain focus as projects shift or expand across disciplines.

The final step, strategy formulation and planning, involves determining how the team will accomplish its goals. Teams assign roles, prioritize tasks, and establish procedures for communication and decision-making. There are three types of planning: (1) deliberate, (2) contingency, and (3) reactive, which differ in timing and focus. Deliberate planning defines structured paths and milestones, contingency planning anticipates potential obstacles and alternative approaches, and reactive planning occurs in response to unexpected challenges during task execution. Teams that engage thoroughly in deliberate and contingency planning are better equipped to adapt through reactive planning later (Mathieu and Rapp, 2009). This flexibility is essential in collaborative research teams, where unanticipated findings, institutional changes, or personnel shifts often require realignment midstream.

Ultimately, transition phase processes serve as the foundation for all subsequent teamwork. Clarifying the mission, specifying goals, and developing strategies create alignment that guides action and adaptation as conditions change. These behaviors are not confined to a single moment in time but recur throughout a team’s lifespan, each cycle refining shared understanding and strengthening collaboration, particularly in long-term scientific collaborations. This iterative nature highlights why transition processes are imperative to science teams, where success depends on sustained coordination across evolving research contexts (LePine et al., 2008).

### 4.2 Action phase processes

Once teams establish their goals and strategies during the transition phase, they shift into action, where plans are implemented and refined in real time. The action phase centers on goal-directed taskwork and involves four interrelated processes: (1) monitoring progress toward goals, (2) systems monitoring, (3) team monitoring and backup, and (4) coordination. These processes occur continuously rather than sequentially, allowing teams to adapt their approach as circumstances evolve. Together, they ensure that teams maintain situational awareness, manage resources effectively, and synchronize efforts to achieve their objectives.

Monitoring progress toward goals involves tracking advancement toward established objectives, assessing what remains to be accomplished, and communicating updates across the team. By sharing progress information at scheduled intervals and through informal exchanges, teams enhance transparency and allow members to adjust their work accordingly. Frequent monitoring increases coordination quality and supports timely responses to emerging challenges, leading to higher performance (Marks and Panzer, 2004; Mesmer-Magnus and DeChurch, 2009). In science teams, where research timelines and deliverables often shift with new discoveries, consistent progress monitoring can help maintain alignment across disciplines and prevent drift from shared goals.

Systems monitoring focuses on evaluating the team’s environment, tools, and resources to ensure they remain sufficient for completing the task. Actively assessing whether systems are functioning as intended allows teams to detect problems early and adjust before disruptions occur (Marks et al., 2001; Mathieu et al., 2008). For example, a team may identify that their chosen data-sharing platform lacks key features and decide to switch to a more effective system, thereby avoiding communication breakdowns. This attention to systems is vital in large-scale scientific collaborations, where technologies, data requirements, and institutional constraints often change over the course of a project.

Team monitoring and backup occur when members observe one another’s workload and provide support as needed, whether by offering feedback, assisting with tasks, or temporarily taking over responsibilities. Regular feedback improves coordination and facilitates learning within the team (Gabelica et al., 2012; McLarnon et al., 2019). These behaviors are particularly important in research environments where expertise is highly specialized. Even when members cannot directly perform each other’s tasks, awareness of others’ progress enables timely feedback and cross-disciplinary problem-solving that sustains overall momentum.

Finally, coordination integrates the outcomes of these monitoring processes by aligning interdependent tasks and managing timing. Effective coordination ensures that work flows smoothly, reduces redundancy, and allows teams to use their time and resources efficiently. Tasks with high interdependence require careful sequencing, while those that can occur in parallel demand clear communication to prevent overlap (Marks et al., 2001). Coordination is often complicated by disciplinary boundaries, asynchronous schedules, and geographic dispersion common to scientific collaboration. Intentional coordination practices such as regular check-ins or shared progress dashboards help bridge these divides and maintain cohesion across distributed collaborators.

Collectively, action phase processes translate planning into performance. They help teams remain agile and responsive to change while maintaining progress toward their goals. For science teams, whose work is characterized by uncertainty and evolving complexity, these processes provide the structure and communication mechanisms necessary to sustain collaboration and ensure that the team’s collective effort moves toward discovery.

### 4.3 Interpersonal processes

Interpersonal processes operate continuously across both transition and action phases, serving as the social foundation that enables teams to function effectively over time. While transition and action processes focus on task-related planning and execution, interpersonal processes capture how teams communicate, manage relationships, and maintain motivation throughout these cycles. They include three primary components: (1) conflict management, (2) motivation and confidence building, and (3) affect management. Together, these processes sustain collaboration by promoting trust, cohesion, and adaptability.

Conflict management involves regulating disagreements through preventive and reactive strategies that preserve team functioning (Marks et al., 2001). Although conflict is often associated with negative outcomes, its impact depends on both the type of conflict and how it is handled. Task conflict, which focuses on differing ideas or approaches, can encourage critical thinking and innovation when managed constructively (DeChurch and Marks, 2001). In contrast, relationship conflict, driven by personal tension or disrespect, is consistently linked to poorer performance and diminished trust (Yuan et al., 2025). Effective conflict management enhances cohesion and helps teams recalibrate SMMs after disagreements (Tekleab et al., 2009). In interdisciplinary research settings, addressing conflict proactively is essential for maintaining psychological safety and integrating differing viewpoints into collective solutions.

Motivation and confidence building refer to the behaviors that foster commitment and reinforce members’ belief in the team’s capability to achieve its goals. Acknowledging both individual and collective contributions strengthens confidence and engagement, which in turn enhances team performance (Hamilton et al., 2017). Sustaining motivation can be challenging in long-term, research-oriented teams where project timelines are extended, and feedback is often delayed. Regular acknowledgment of progress, along with visible alignment between individual efforts and team outcomes, helps maintain energy and focus over time.

Affect management involves regulating the team’s emotional climate before, during, and after demanding or stressful events. Teams that manage emotions effectively are better equipped to navigate setbacks, reduce tension, and maintain constructive interactions (Jiang et al., 2013). Affect management supports resilience and helps ensure that emotional responses do not hinder problem-solving or collaboration, which is a vital function for teams navigating uncertainty, competing priorities, and intermittent progress. A positive affective tone also reinforces trust and openness, enabling members to exchange feedback and engage in creative dialogue.

## 5 Evaluation of models

### 5.1 Similarities between the Team Process and the Transdisciplinary Team Development models

In selecting the Marks et al. (2001) and Hall et al. (2012) models to develop our framework, we identified two key characteristics they share. First, both models represent how teams move across logical and time-oriented patterns. In Hall et al. (2012), teams begin with an initial idea that progresses to conducting the necessary research to generate findings that are either applied to the original or a new context. The stages they move through are cyclical, requiring completion of one stage prior to moving to the next. Similarly, the team process framework also dictates that teams move from transition to action phases, with transition phases occurring before the team engages in activity. Coordinated action requires the team to discuss the anticipated goals and work approach. Together, the nature of these models represents how literature in both science teams and organizational teams recognizes forward progression, moving from ideas to action.

The second key consideration in adopting both models is that even though stages or processes occur before others begin, both models propose that teams may return to a prior stage or process. Science as a field requires much trial-and-error to develop sound experiments to address research questions. The Hall et al. (2012) model specifically states that teams may need to return to a previous stage if they have experienced potential conflict, failure, or a redefinition of goals. Thus, before the team disbands or initial goals are achieved, they may encounter multiple recursive steps to previous stages. Likewise, according to the team process model, teams may move from a transition phase into task execution, then recognize the need to reconceptualize the work or address unanticipated challenges that emerge, and they have to go back to a transition phase. Moving fluidly through different stages or processes creates natural alignment between the two models, providing a path for mapping the team processes that occur as science teams move forward to future stages or take a step back based on the specifications from the Marks et al. (2001) model.

### 5.2 Differences between the Team Process and the Transdisciplinary Team Development models

As noted above, the two selected models share substantial overlap in how they present the ways in which teams move throughout time. However, a key difference between the models is in how explicitly they discuss team processes across stages. In the Hall et al. (2012) framework, teams are described as encountering several interactions—such as defining goals, engaging in conflict, and having to create shared language and mental models—but these processes are not systematically elaborated or defined within each stage. In contrast, Marks et al.’s (2001) model specifies what teams are doing at particular moments in time, detailing transition, action, and interpersonal processes as they unfold across performance cycles. However, this model is not stage-specific and does not require a specific process to occur at set development stages. Instead, it assumes a general temporal order in which transition processes precede action processes. The difference between these models surfaces how each of these frameworks is rooted in different, but complementary levels.

### 5.3 Selecting the Team Process and the Transdisciplinary Team Development models for our framework

Ultimately, the Hall et al. (2012) and Marks et al. (2001) models were selected because they allow for micro-level team processes that happen in transition, action, and interpersonal phases to be easily mapped to broader macro-level stages that science teams engage in. In selecting these two models, we can clearly delineate not only which team processes occur in each stage and which behaviors are most critical for team success, but also the processes these teams engage in when they must move backward to a prior stage. As both models account for dynamic and temporal changes throughout the team’s lifecycle, we can articulate the defining and salient processes at each stage.

Other team models, such as Tuckman’s (1965) stage-based model, do not capture the dynamic changes that occur in science teams. Although foundational, this early model offers a limited conceptualization of how teams interact over time, specifying broad stages, as compared to specific behaviors. The I-M-O-I and systems-based team model frameworks from Ilgen et al. (2005), Mathieu et al. (2008, 2017b) also provide a macro-level conceptualization of teams, showing how teams are situated within multilevel structures and indicating there are additional emergent elements that connect team inputs and outputs. However, they have limited insight into episodic or moment-to-moment behaviors within teams as they advance through a performance cycle. As a result, such frameworks are better suited to describe how inputs, mediators, and outputs are connected and span multiple levels, rather than providing a detailed account of team processes within stages.

In the team science literature, few models exist, with the work of Stokols et al. (2008) being some of the earliest work to begin exploring an ecological perspective on science teams and the context surrounding them. These frameworks emphasize the broader external factors that influence teamwork, accounting for antecedents, team processes, and outcomes (i.e., teams that are not face-to-face), and are helpful for examining science teams from a macro level. While Hall et al.'s (2012) model is still a macro-level model, it emphasizes the specific steps science teams are completing within the four broader stages it presents. Together, the choice to integrate Marks et al.’s (2001) and Hall et al.’s (2012) models is clear. When combined, this integration offers a more rigorous, comprehensive, and temporally precise framework than either model could alone. By moving beyond broad categorizations, our integrated framework specifies *which* teamwork processes matter at developmental phases, providing a level of precision and temporal alignment that existing team and science team models do not fully capture.

## 6 Introduction to the framework

The Marks et al. (2001) and the Hall et al. (2012) models are complementary and can be mapped onto each other, describing the cyclical and recursive nature of science teams. A key foundation of both frameworks is their temporal orientation. Transition processes precede action processes, and the output of action feeds future transitions. Likewise, teams progress through Development, Conceptualization, Implementation, and Translation. They may work iteratively, returning to earlier phases to address unforeseen needs. Together, these two models illuminate when teams engage in specific processes and promote effective performance behaviors.

Marks et al.’s (2001) framework provides an iterative structure for how teams operate in a dynamic landscape, often working through multiple transition and action processes that reframe and reorient the task. Although it has been extended to a broader range of team types (Mathieu et al., 2008), its application to the complex, distributed, and phase-sensitive work of science teams remains limited. Like the team process model, Hall’s four-stage model is not strictly linear, and teams may adjust and update plans throughout. It also allows scientific teams to function recursively, whereby the output of one project feeds into another new application. Such recursion is particularly common in labs or centers focused on research translation that sustain long-term collaboration.

Combining the team process and four-phase models creates a more nuanced view of how scientific teams operate and the behaviors they encounter at different phases. The team process model provides descriptive behaviors of what occurs as teams develop and implement ideas. The four-phase model aligns with the team process model by indicating when teams are in transition or action phases as they move from the initial idea to its translation. Additionally, the four-phase model describes behaviors and emergent states such as psychological safety, while the team process model provides labels for each phase.

**Figure 1:**
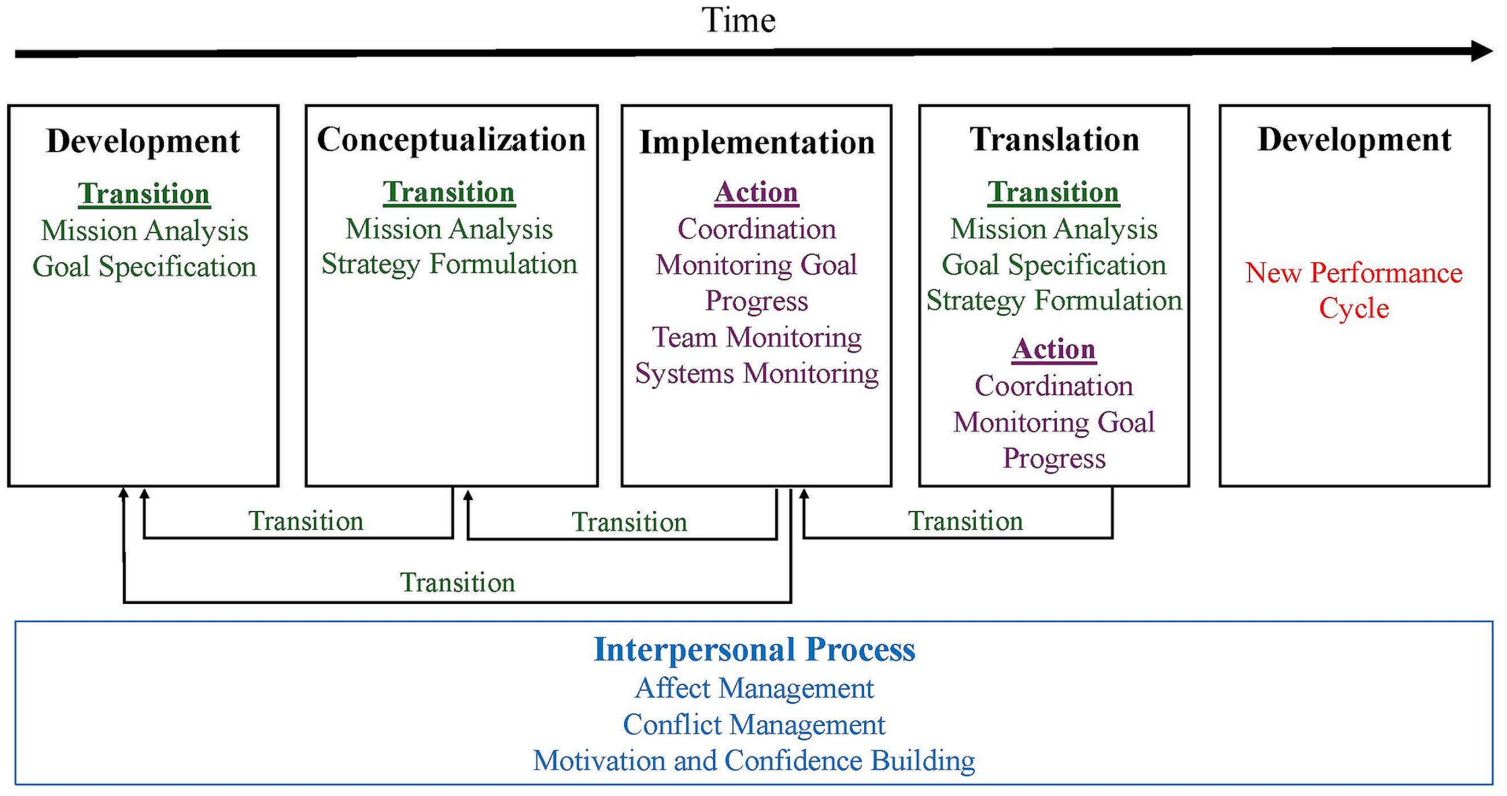
An integrated framework for science team processes across project phases. The figure depicts the authors’ integrative framework of science team processes across four stages. Content is adapted from Hall et al. (2012) and Marks et al. (2001).

In integrating Hall et al.’s (2012) four-phase model with Marks et al.’s (2001) temporally based process model, we followed a conceptual integration approach grounded in theoretical alignment rather than a formal systematic review. Specifically, we examined the focal activities and temporal location of each Hall phase and mapped them onto Marks et al.’s (2001) transition, action, and interpersonal processes based on their primary behavioral focus and function in the performance cycle. The processes emphasized at each phase are those that are both theoretically central for science teams and recurrent in existing empirical descriptions of science teams, ranging from unidisciplinary to transdisciplinary collaborations. The resulting framework highlights the importance of transition processes and how early planning supports downstream coordination. A shared feature of our framework and Marks et al.’s (2001) is the reliance on interpersonal processes throughout each stage of the performance cycle. By integrating these two frameworks, we add to the psychology and team science literature by proposing a new conceptualization of how team processes unfold within a staged model. The full framework is presented in Figure 1.

## 7 Presentation of the framework

In this section, we ground our presentation in examples and integrate the two models from a transdisciplinary team perspective, given that Hall et al. (2012) explicitly address processes in transdisciplinary teams. The stages and processes that many science teams go through are built on this scaffold, but it should be emphasized that the salience of the processes at each stage varies with the composition of the science team. We then apply the framework to other science teams to illustrate how the combined approach extends beyond transdisciplinary teams.

### 7.1 Development

In the Hall et al. (2012) model, the Development stage surfaces a research question that an initial group of advocates finds relevant. Through discussion of the idea, the initial group brings together additional relevant members, often filling complementary roles and topical knowledge required to address the proposed problem. The functional diversity that members bring is beneficial for viewing the problem from multiple angles, but it also challenges the broader team because members may hold differing motivations and goals for the project (Chen et al., 2025). Thus, in the Development stage, goal specification is the defining process teams engage in. Specifically, the team works to develop a shared understanding of the problem and its associated goals. Early stages of mission analysis also occur toward the end of Development, as the conversation about goals shifts into a discussion of what resources are available and how the goals will be executed. The Development stage clearly represents a transition process through the definition of a shared goal and the prioritization of subsequent goals that team members individually hold.

Emphasis on establishing communication norms and minimizing conflict is critical to supporting goal specification. Science teams that have greater disciplinary differentiation and specialization may introduce barriers to describing and understanding the problem across divergent perspectives. Thus, minimizing discipline-specific jargon and establishing a shared language for discussing potential paths forward facilitates the development of a SMM and a collective understanding of what must be addressed at a generalized level, irrespective of disciplinary boundaries (Fiore et al., 2013; Mathieu et al., 2017a; Thompson, 2009). A shared understanding reduces the potential for misunderstanding and conflict, thereby fostering clearer and more inclusive communication and cohesion, both in the early stages of collaboration and as the team continues to evolve.

Furthermore, competing goals, as noted by Hall et al. (2012), may generate intergroup conflict over prioritization, privileging one discipline over another and thereby diminishing the collective benefit required to achieve a goal that transcends disciplinary boundaries. Careful attention to mitigating such conflict, while recognizing the value of the team as a whole and the ways in which individual expertise contributes rather than competes with others, establishes a strong foundation for subsequent strategy development and task implementation (Behfar et al., 2008). Role clarity emphasizes the specific contributions of each team member. In transdisciplinary science teams, it is especially important to draw connections showing how these roles are complementary and interdependent. Specifying these functions at the outset of the team, and as new members are added, promotes team cohesion, demonstrating that without each role, gaps would emerge and that the team has been deliberately assembled to address the problem collectively (Lynn and Kalay, 2015). The use of team charters has been highlighted as a formal mechanism for clarifying roles and responsibilities, as well as for establishing communication preferences and protocols (Rapp, 2025).

Although conflicting goals are to be anticipated, creating a psychologically safe environment from the beginning can help recognize and value ideas and feedback that differ from what members are familiar with. Specifically, when new conceptualizations of the goal are presented, engaging in dialogue to understand members’ reasoning demonstrates a desire to learn and build on one another’s work (Dietl et al., 2023; Edmondson and Roloff, 2008). Expressing differing opinions early, while the problem set is still being defined and goals are being established, can positively shape later stages by allowing ideas to be refined and assessed for potential consequences and gaps. It also sets the tone for continuing to operate in a psychologically safe environment and enables members to build comfort and confidence, often with collaborators they may not have worked with previously, a dynamic often described as swift trust (Blomqvist and Cook, 2018; Elms et al., 2023). A unique feature of these transdisciplinary teams is that prior experience working together may be minimal, and members have specialized knowledge. Therefore, members need to believe from the outset that their colleagues are adept and can provide expertise to the problem set, establishing cognitive trust.

Ultimately, entering the Development phase with an appreciation for the individual expertise that informs the collective effort fosters trust and creates a foundation rooted in team norms and shared understanding (Brown et al., 2023). Given the often-high degree of knowledge and role diversity typical of transdisciplinary science teams, the development of a charter facilitates an understanding of both unique and shared expertise, fostering a collective framework for how members will collaborate and contribute to the team’s overarching goals. Team charters can also specify communication practices, including the tools to be used and the expected frequency of interactions (Rapp, 2025). Through this process, communication norms are explicitly established and serve to guide team functioning. Importantly, as team composition changes or the nature of the scientific problem shifts, the charter should be revisited and revised to ensure continued alignment with evolving goals and membership.

#### 7.1.1 Propositions

*Proposition 1:* During the Development phase, higher quality team charters during transition processes will be associated with greater role clarity and more effective communication, which in turn will be associated with lower relationship conflict.

*Proposition 2:* During the Development phase, greater use of shared, non-discipline-specific language in interpersonal interactions will be positively associated with the development of shared mental models.

### 7.2 Conceptualization

In Transdisciplinary teams, after a shared understanding of the problem and goals has been agreed upon, the specific research questions, hypotheses, and methods must be developed. The discussions at this stage align with Marks et al.’s (2001) mission analysis and strategy formulation and planning, again representing the transition processes the team engages in. As the research design begins to take shape, it requires maintaining a clear and deliberate linkage between the research question, the selected approach, and the prioritized goals. In translating the goal into an actionable plan, multiple perspectives yield alternative routes that must be evaluated. For example, the need for additional expertise may arise as hypotheses develop, and the team may recognize that another member is needed to fill gaps. Evaluating the expertise currently on the team and what is still needed reflects mission analysis by assessing available resources. Resource analysis then informs how those resources will be used to advance specific goals. For transdisciplinary teams, the resources required to conduct research are facilitated by the team’s composition, as members have access to diverse materials that can be combined for a stronger research endeavor.

Building on early efforts in goal specification and mission analysis, the Conceptualization stage primarily focuses on strategy formulation to establish a cohesive approach that aligns across varied disciplines. Each discipline brings methods for constructing the research plan. The breadth of knowledge allows optimization by selecting and combining methods from different disciplines and roles (e.g., applied versus academic settings) that are complementary and might not have been considered without the team’s diverse composition.

The strategy develops through iterative discussion of individual contributions that can be integrated into a collective approach, ultimately building a full SMM and a TMS. Disclosing expertise and knowledge allows members to identify who holds unique, differentiated knowledge and where knowledge is shared within the team. In effect, the team develops a TMS that identifies who knows what and how new information will be retrieved, encoded, and shared as the project moves forward (Lewis and Herndon, 2011). Comprehensive strategy formulation prior to engaging in the research process helps reduce the risk of backtracking. Thus, a strategy that fully leverages differentiated knowledge and methods minimizes the limitations of any single approach and provides contingencies for deviations from the main plan.

As teams develop the overarching research plan, it is essential that they establish clear decision-making procedures that specify how decisions will be made and who will be involved in each step of the implementation process. Updating the team charter to include an explicit outline of these decision-making protocols helps to foster a shared understanding and collective mindset among team members (Bennett et al., 2013; Jacobs et al., 2015; Marks et al., 2001; Sixsmith et al., 2020). Establishing such protocols also creates pathways for healthy dialogue between team members when there is a decision point, through establishing norms and structured scripts that guide the decision-making process. This proactive approach supports transparency, minimizes misunderstandings, and strengthens the team’s capacity to navigate challenges collaboratively.

#### 7.2.1 Propositions

*Proposition 3:* During the Conceptualization phase, more explicit strategic planning and decision-making protocols in transition processes will be positively associated with transactive memory systems, which will be associated with lower relationship conflict.

*Proposition 4:* During the Conceptualization phase, higher levels of affect management during interpersonal processes will be positively associated with psychological safety, which will in turn be associated with more critical evaluation of ideas.

### 7.3 Implementation

As teams advance into the Implementation stage, the strategy formulation, mission analysis, and goal specification completed during Development and Conceptualization should guide action processes as the team carries out the research plan. During Implementation, the team answers the research questions and conducts the agreed-upon methods. Teams have moved past analyzing specific approaches and are now shifting their attention to completing the research. The specific action processes hinge on interdependence between team members. When work is closely coupled, coordination demands are higher; when tasks can proceed more sequentially, they are lower. Nevertheless, coordination remains critical in transdisciplinary teams, given varied knowledge bases and limited prior joint experience (Wong-Parodi and Strauss, 2014). When knowledge variation is high, understanding what comes next in each team member’s process is not readily apparent. Work in earlier stages should have increased awareness of counterparts’ workflows, yet as the research progresses, deviations from the original plan can occur, shifting how members need to coordinate and requiring reliance on established contingency plans.

A distinctive feature of teams with varied expertise is how monitoring processes and backup behaviors unfold. For instance, if a team consists of one individual per discipline, other members are unlikely to provide direct backup on specialized tasks. Feedback may instead take the form of general suggestions to improve handoffs or alternative ways to structure work (Porter et al., 2003). As teams grow more diverse with individualized roles, the SMM and the TMS developed earlier help to bridge gaps during execution. Monitoring still occurs, but help is provided differently: members offer broader feedback rather than hands-on assistance when cross-training is limited. When direct assistance is required, additional time for familiarization and brief training may be needed to enable effective backup.

Even when cross-domain backup is limited, teams still need to track progress against earlier goals. Regular check-ins to assess whether each task is moving in the expected direction and timeline can partially substitute when cross-domain backup is constrained. Communication plans established during transition phases set expectations and cadence for these check-ins. Teams should also address problems in the operating environment: if resources are being consumed faster than anticipated or collaboration tools are ineffective, systems monitoring should prompt adjustments to avoid disruption. Proactive detection of issues (current or potential) can indirectly support backup behaviors and provide useful feedback even without deep knowledge of others’ tasks. These behaviors also help promote task-related trust within the team (Colquitt et al., 2007; Feitosa et al., 2020), as team members know the work is being done and they can rely on each other’s expertise to complete their necessary tasks.

#### 7.3.1 Propositions

*Proposition 5:* During the Implementation phase, higher quality action processes, including progress monitoring and coordination, will be positively associated with team trust, which will in turn cause lower levels of relationship conflict.

*Proposition 6:* During the Implementation phase, higher levels of motivation-building behaviors during interpersonal processes will be positively associated with individual and team efficacy.

### 7.4 Translation

Once a transdisciplinary team has completed and refined the research conducted during Implementation, the results can be applied to the problem identified in Development or extended to related applications. For example, a team addressing a novel public health issue (e.g., COVID-19) may first apply insights to the original problem, then recognize that similar methods could inform responses to other highly contagious illnesses. Pursuing a new application typically returns the team to Development and Conceptualization to clarify goals, stakeholders, and methods for the new context before proceeding.

During Translation, transdisciplinary teams engage in action processes that turn findings into solutions. Coordination is central to ensuring translation activities proceed in a deliberate sequence, reduce redundancy, and align how different disciplines contribute to a shared plan. Monitoring progress tracks adoption of milestones, partner engagement, and early impact indicators. Systems monitoring attends to practical enablers and constraints such as organizational readiness, policy requirements, data governance, and dissemination platforms. Team composition may shift to include implementation partners and knowledge brokers, including practitioners, policymakers, community champions, and technology transfer staff who bridge science to real-world use.

Nested within Translation are transition processes that define how findings will move into practice. Goal specification at this stage focuses on the dissemination and adoption of objectives rather than data collection. Expanding efforts means that transdisciplinary teams have to identify target users, contexts, timelines, and success metrics, and ensure that these outcomes remain tied to the mission articulated in Development. Strategy formulation outlines pathways for implementation and scale, clarifies decision rights, allocates resources, and establishes plans for authorship, intellectual property, and credit.

Further, these teams sometimes face a choice between concluding the performance cycle by applying findings only to the original problem or launching a related line of work. Differing preferences can create friction. A psychologically safe environment supports open discussion of tradeoffs and helps members understand why certain Translation paths are prioritized. Members who remain focused on the primary goal may choose to complete implementation of solutions and then rotate off, while others re-engage in a new cycle aimed at a related application. Transparent decisions and timely acknowledgment of contributions reduce the risk that conflict undermines cohesion.

Regardless of the path teams choose to pursue, having a structured debrief in which the team walks through successes, failures, and areas for improvement can help conclude the action processes they were engaged in and provide a summary of their efforts (Salas et al., 2018). Debriefs should be held at the beginning of the Translation stage, as the discussion can provide useful next steps for applying the findings to the original problem or in a new context. Strong debriefs can help to strengthen the future collaboration of this team or new teams.

Even if new avenues for research are not pursued immediately, teams can serve their long-term interests during this stage by intentionally planning for sustained collaboration. Discussing potential directions for future research and identifying shared interests among members establishes a foundation for ongoing partnerships (Shukla, 2024). Creating a repository of next steps, along with noting who is interested in specific follow-up efforts, helps preserve institutional knowledge generated during this performance cycle, and facilitates the re-engagement of the team after periods of inactivity or as new members are recruited for succeeding research cycles.

Trust built during earlier phases carries forward. Translation often introduces new partners and public visibility, which raises stakes for reliability and fairness. Consistent follow-through on commitments, open attribution of contributions, and equitable access to opportunities reinforce cognitive and relational trust. Outcomes from Translation then feed the next performance cycle, either scaling the original solution or launching a related effort. The team typically returns to Development and Conceptualization to realign goals, roles, and methods for the new context, which preserves the cyclical and recursive nature emphasized by Hall et al. (2012).

#### 7.4.1 Proposition

*Proposition 7:* During the Translation phase, structured team debriefs at the end of performance episodes will be positively associated with shared mental models, which will, in turn, increase trust and psychological safety in subsequent phases.

### 7.5 Interpersonal processes

Interpersonal processes within all science teams are continuously occurring. Consistent with Marks et al. (2001), these processes are essential across both transition and action phases and are represented similarly in our framework, spanning the entire cycle from Development to Translation. Broadly, as science teams progress through these stages and move between transition and action processes, the ways in which they engage in conflict management, motivation and confidence building, and affect management vary according to the specific demands and context of each stage.

#### 7.5.1 Conflict management

Conflict is an inevitable aspect of all teams. However, transdisciplinary teams may notice that conflict is amplified by greater differences in knowledge and experience. During the Development stage, these teams need to work to establish a shared understanding of the problem and its goals while navigating individual objectives to align around the team’s broader, collective mission (Hall et al., 2012).

Conflict may also emerge early in these teams when deciding who should belong on the team. If the current composition of the team does not fulfill the necessary expertise to solve the problem of interest, additional team members may need to be added. The addition of these new members may temporarily increase task conflict as the research goals and plan need to be updated and integrated with the new team members (Behfar et al., 2008).

As the team is actively carrying out the research plan, conflicts can occur if team members are not coordinating well. Specifically, for transdisciplinary teams, the specialization of each team member increases the difficulty of supporting research processes outside each team member’s specialty. Teams that coordinate effectively during action phases reduce the amount of task conflict they may experience as they know who is doing what, how it is getting done, and how their role fits into the timeline (Lewis and Herndon, 2011). Conflict increases when these teams do not have a strong understanding of when they are supposed to be working and who is relying on their information to perform their part (Jehn, 1995). When translating the work, conflict may surface around which application to prioritize first, how to allocate limited resources, or how credit will be handled (Sixsmith et al., 2020). Clear decision criteria, revisiting authorship and credit norms, and structured prioritization tools help keep disagreements task-focused.

Without careful, quick, and constructive mediation, task conflict can escalate into relationship conflict, undermining the task and, more seriously, affecting team members. In severe cases, such conflict may prompt a team member to withdraw from the project, necessitating at minimum a realignment of roles or, at worst, a significant delay as a new member is recruited, onboarded, and brought up to date, potentially introducing further disagreement in the current process. Conflict may also affect the whole team by reducing morale and motivation, slowing progress, and contributing to feelings of isolation through the development of in-groups and out-groups (Simon, 1992).

Conflict should be anticipated, and transdisciplinary teams can prepare for it. It is important to establish communication norms for how team members express dissenting opinions before conflict occurs. Setting norms early can help establish the tone of productive communication. When conflict occurs, taking quick action to identify where processes were interrupted and providing solutions can help reduce negative outcomes such as reduced productivity and trust (Shrum et al., 2001).

#### 7.5.2 Motivation and confidence building

Throughout the research endeavor, sustaining motivation and confidence can help to generate cohesion and the desire to keep working, even during setbacks. Motivation and confidence can be sustained through short impact updates, brief milestone celebrations, stakeholder testimonials, and regular “wins” retrospectives that connect individual contributions to external outcomes. In stages such as Development and Translation, motivation will naturally be higher as the team begins the research and takes the next steps to apply the findings. However, motivation and confidence-building can be most impactful toward the middle of the science team’s efforts. For example, if during Conceptualization the team has sifted through numerous approaches and has not landed on a collective way forward, this can feel defeating and lead to a drop in motivation (Bandura, 1997; Bandura, 2006). Further, if teams have reached the Implementation phase and initial processes are not working as expected, this can also lower motivation.

Motivation can be renewed by recognizing and reengaging in behaviors that have previously led to positive outcomes. One way transdisciplinary teams can achieve this is through revisiting and replicating the processes that successfully facilitated moving from an earlier stage. Reflecting on prior achievements may allow the team to identify which practices, communication strategies, or decision-making approaches contributed to earlier successes (Schwarzer and Jerusalem, 1995). This process allows the team to internally reflect on how they can create forward progression, even within a single stage, if they are having trouble addressing a failed task in the Implementation phase.

#### 7.5.3 Affect management

An underlying assumption about many science teams is a deep individual and collective desire to help answer questions with the potential to yield real-world implications. This desire can elicit strong emotions within the team, which should be monitored. It is important to allow negative emotions, but not to allow them to escalate or persist. In transdisciplinary teams, affect management is most important during the Conceptualization, Implementation, and Translation stages, as strong emotions may be expressed within the team and can impact the performance cycle. For example, during the Implementation stage, if team members working in parallel encounter problems that block handoffs to the next step, diagnosing the source of error promptly prevents downstream waste. Back-to-back conflicts can destabilize team affect. Acknowledging inevitable growing pains and framing setbacks as learning opportunities can ease negative feelings and prevent contagion (Barsade et al., 2018). Although the team may be experiencing frustration, acting quickly to address conflict and work plan deviations helps mitigate the escalation of negative affect that could have longstanding implications. Strategies including reflection debriefs, cadence resets, and appreciation rituals help maintain a constructive climate and preserve psychological safety so concerns and alternatives can be voiced and addressed, and help to regulate the team’s affect (Salas et al., 2018; Zhang and Zhang, 2014).

### 7.6 Moving back to a previous stage

Hall et al.’s (2012) model is cyclical while also allowing backward movement through the stages when teams need to restructure plans or redefine processes. A team can step back one stage or even two, for example, from Implementation to Development. In the team process model, any return is a transition episode because previously set mission, goals, and strategies are being reconsidered. For instance, if a transdisciplinary team advances from Development to Conceptualization and then discovers that a priority goal is missing or incomplete, the appropriate response is to return to Development, reopen goal specification, and then proceed again with strategy formulation.

Backward movement requires concrete behaviors aligned with the earlier phase. If goals change and new members are added, the team must onboard them, clarify roles, and update communication norms to reflect the new composition. Mission analysis should be refreshed to capture revised constraints, resources, and stakeholder needs. Shared mental models and the TMS need to be re-baselined, so everyone understands who holds which expertise and how information will be encoded, retrieved, and shared when action resumes. These steps re-establish alignment and protect coordination when the team re-enters execution.

Since backtracking signals that something has not worked as intended, interpersonal processes are especially important. Conflict management helps keep debates task-focused while methods and goals are revised. Motivation and confidence-building counter the energy dip that could follow a return to the drawing board, particularly if Implementation had already begun. Affect management sustains psychological safety so members can voice concerns and propose alternatives without blame, which is critical when membership changes disrupt social cohesion, and trust must be rebuilt (Poliseli and Leite, 2021).

Stepping back is not a failure; it is disciplined learning. A pilot conducted during Implementation may reveal design flaws that would blunt the impact in Translation. Returning to Conceptualization to revise the protocol can prevent wasted effort and increase the likelihood of meaningful application. Although the short-term effect may be a temporary drop in morale, a clear rationale for the reset, rapid re-planning with visible milestones, and recognition of contributions during the pivot can restore positive affect and sustain momentum into the next cycle.

Overall, our framework shows how the Hall et al. (2012) and Marks et al. (2001) models can be integrated to depict team processes occurring at each stage of a team’s lifecycle. We specifically focus on the initial application of this framework to transdisciplinary teams. In doing this, we highlight the challenges and opportunities these teams may encounter as they collaborate, along with the behaviors that may be helpful at each stage. We also discuss how teams may not always move in a cyclical manner, having to go back to a prior step, and acknowledge that transition processes are critical in these times of realignment. Figure 1 presents a graphical model of our framework. We also provide a table that illustrates how the two models align with the explicit team processes identified by Marks et al. (2001) and the stages outlined by Hall et al. (2012). Table 1 summarizes the key components of each model and demonstrates how they are integrated within our proposed framework. Each stage-specific proposition can be noted in Table 2, along with example measures for future testing.

**Table 1:** Conceptual integration of Hall et al. (2012) and Marks et al. (2001) into a new framework.

Hall et al. (2012) Stages	Key features of stage	Marks et al. (2001) Processes	Integration of frameworks
Development	Shared mission and goal setting Create psychological safety Start organizing the team	*Transition* Goal specification Mission analysis *Interpersonal* Conflict management	Teams are developing the problem of interest and, in doing so, begin to understand what the broad achievement is and the specific goals that need to be achieved.
Conceptualization	Development of research questions and scientific approach Creation of shared mental model Building a transactive memory system	*Transition* Mission analysis Strategy formulation *Interpersonal* Conflict management Motivation and confidence Affect management	With established goals, the team begins to outline the necessary steps to reach each goal and outline the strategy and performance required.
Implementation	Engage in the specified approaches Refine approach as needed Manage conflict	*Action* Coordination Team monitoring Monitoring goal progress Systems monitoring *Interpersonal* Conflict management Motivation and confidence Affect management	The team begins to work toward the outlined activities. During these working periods, they are updating each other on task progress, conflicts that occur, and what resources are effective or not for collaboration efforts.
Translation	Apply findings to the initial problem set and/or determine a new application of findings to a different, but related problem set Created shared goals around translation Create a shared strategy for achieving the outlined goals	*Transition* Goal specification Mission analysis Strategy formulation *Action* Coordination Monitoring goal progress *Interpersonal* Conflict management Affect management	The team culminates the research findings, wraps up the research, and begins creating goals and strategies for applying solutions to the problem set based on the findings. Teams can also recognize how these findings may apply to another, related problem and begin transitioning and developing a plan for creating a new research venture to address the problem through a new lens.

Content adapted from Hall et al. (2012) and Marks et al. (2001) and integrated within the authors’ conceptual framework.

**Table 2:** Stage-specific propositions and illustrative measures across team process phases.

Stage (Hall et al., 2012)	Propositions	Examples of validated measures for empirical testing
Development	Proposition 1: During the Development phase, higher quality team charters during transition processes will be associated with greater role clarity and more effective communication, which in turn will be associated with lower relationship conflict. Proposition 2: During the Development phase, greater use of shared, non-discipline-specific language in interpersonal interaction will be positively associated with the development of shared mental models.	Communication (Hoegl and Gemuenden, 2001) Conflict (Jehn, 1995) Shared mental model (Mathieu et al., 2017a)
Conceptualization	Proposition 3: During the Conceptualization phase, more explicit strategic planning and decision-making protocols in transition processes will be positively associated with transactive memory systems, which will be associated with lower relationship conflict. Proposition 4: During the Conceptualization phase, higher levels of affect management during interpersonal processes will be positively associated with psychological safety, which will in turn be associated with more critical evaluation of ideas.	Psychological safety (Edmondson, 1999) Transactive memory systems (Lewis and Herndon, 2011) Conflict (Jehn, 1995)
Implementation	Proposition 5: During the Implementation phase, higher quality action processes, including progress monitoring and coordination, will be positively associated with team trust, which will in turn cause lower levels of relationship conflict. Proposition 6: During the Implementation phase, higher levels of motivation-building behaviors during interpersonal processes will be positively associated with individual and team efficacy.	Collective efficacy (Bandura, 1997) Individual efficacy (Schwarzer and Jerusalem, 1995)
Translation	Proposition 7: During the Translation phase, structured team debriefs at the end of performance episodes will be positively associated with shared mental models, which will, in turn, increase trust and psychological safety in subsequent phases.	Trust (For a collection of all trust measures, see Feitosa et al., 2020)

The table presents example construct measures for empirically testing the proposed relationships, adapted from each stage of the Hall et al. (2012) model. Propositions reflect the authors’ original framework, and cited measures are illustrative examples drawn from prior literature.

## 8 Extending our framework across team types

As has been established in the literature, scientific teams also vary in their configuration, ranging from unidisciplinary to transdisciplinary teams, each representing different compositions of team members and varying substantially in the degree of cognitive integration required (Bates et al., 2023; Hall et al., 2012; Love et al., 2022; Salazar et al., 2012; Stokols et al., 2012). In discussing our framework we first applied it to transdisciplinary teams. Now, we extend the application of our framework to demonstrate its utility in other common science team configurations, highlighting the similarities and differences these teams may face in each stage and when phases may be more salient.

### 8.1 Unidisciplinary teams

Science teams can also be comprised of members from a single discipline, such as a team of computer scientists working to create new software applications (Zeiss and Steffen, 1998). In unidisciplinary teams, the Development stage is typically shortened (as compared to teams with greater disciplinary diversity) because there is a reduced need for transition processes as members have shared knowledge through similar training and expertise. There may be small learning curves, as each member of the team may have knowledge of different aspects of the particular discipline. However, at large, the high degree of similarity benefits the team through reduced cognitive efforts to understand multiple disciplines and connect their role to others.

Conceptualization in these teams will also be easier to navigate as the theories that drive their research should stem from a single field. Although all team members are from the same discipline, it is not to say that other prior experiences they have will not come into play and bring forth concepts outside of the main discipline. Largely, unidisciplinary teams may experience less conflict at conceptualization because they are familiar with common approaches to structuring the research.

Coordination, feedback, and backup behaviors will be easier in these teams too. Even if team members are working on different components of the research and are not working concurrently, if a problem arises, other team members can more readily fill in and provide feedback due to their shared awareness and knowledge. Monitoring is more facilitative for these teams due to understanding what should be completed at agreed-upon check-in points.

Finally, during Translation, unidisciplinary teams may find it more challenging to extend their results beyond their own knowledge and discipline. The benefit of having a team with a multidisciplinary makeup is that findings can impact more than one area. With unidisciplinary teams, additional effort is required to extend results beyond their unique knowledge. Interpersonal processes will be most critical here as the unidisciplinary team works to generate novel applications of their work. Remaining open-minded and supportive of ideas that seem outside disciplinary expertise can foster future collaborations, which may be interpreted as risk. Managing conflict and emotion can help to reduce the fear of discussing such ideas, as the team is just exploring new opportunities. Based on our framework, unidisciplinary teams experience effectiveness earlier on and may experience more conflict and constraints as they are translating their work.

### 8.2 Multidisciplinary teams

Multidisciplinary teams are characterized by collaboration across different disciplines, though members typically operate within the boundaries of their respective fields (Fawcett, 2013). Knowledge from each discipline is shared, but integration occurs to a lesser extent than in interdisciplinary or transdisciplinary teams. While ideas and approaches are exchanged among members, the focus is not on generating new knowledge that transcends disciplinary boundaries. As a result, the level of interaction among multidisciplinary team members is generally less frequent, allowing individuals to work relatively independently, in parallel, requiring limited coordination and fewer joint transition processes (Stokols et al., 2012).

Our integrated framework highlights how multidisciplinary teams function across stages. During the Development stage, these teams often follow patterns outlined in the Hall et al. (2012) model, such as identifying the problem and establishing collective goals. However, because multidisciplinary teams are not focused on creating new, cross-disciplinary knowledge, they may experience fewer conflicts arising from competing disciplinary priorities. When team members have previously collaborated, pre-existing professional relationships foster a baseline level of trust. However, similar to transdisciplinary teams, swift trust ensues and is established quickly based on role expectations and professional competence to support early coordination and role clarity, especially with the addition of new team members (Blomqvist and Cook, 2018).

The Conceptualization stage operates similarly across transdisciplinary and multidisciplinary teams, as the development of SMMs and TMS can facilitate the research process. However, because multidisciplinary teams place less emphasis on generating collective knowledge that integrates multiple perspectives, they may progress through this phase more efficiently, particularly when strong SMMs have already been established. During the Implementation stage, research activities in multidisciplinary teams are typically carried out independently, with limited interaction or coordination points throughout the process. Team members approach the research from their respective disciplinary lenses, contributing findings that are later synthesized or exchanged as needed. While coordination remains an important team process, it is less pronounced due to the higher degree of independence among members and the clear delineation of roles based on disciplinary expertise. For example, in multidisciplinary public health collaborations, statistics, epidemiologists, public health specialists, and behavioral scientists may have an initial meeting to develop shared awareness, but often work independently during Implementation and then culminate their discipline-specific findings in the Translation stage (Stokols et al., 2012).

Once the identified research question has been answered, some members of multidisciplinary teams may find that the task has been completed and that Translation is not needed. However, multidisciplinary teams can further apply their findings by presenting their work to the intended audience. Engaging the audience beyond what results are published (i.e., through conversations with stakeholders) actively extends their findings to people, and future applications can potentially be identified. Finally, within multidisciplinary teams, as they are preparing to disseminate their work, the team itself may identify additional pathways to continue related streams of research or applications to real-world settings, especially as the research may have shifted from what was initially set out. Overall, multidisciplinary teams may find that during Implementation and Translation, they execute their work in parallel and may struggle to find opportunities to apply their findings.

### 8.3 Interdisciplinary teams

Interdisciplinary teams are more integrated than multidisciplinary teams, as they aim to create shared knowledge across disciplines within a collective framework. They have a higher need to negotiate methodological alignment and shared goals, increasing both transition and interpersonal processes (Stokols et al., 2012). They differ from transdisciplinary teams in that they do not develop entirely novel frameworks that transcend disciplinary boundaries (Hall et al., 2012). Instead, interdisciplinary teams focus on addressing questions that require the exchange of discipline-specific information and the synthesis of that knowledge into a model that advances a prioritized research problem (Fiore, 2008). These teams may form to address both short- and long-term challenges, and the nature of these problems influences how they engage in each phase of development.

During the Development stage, research problems are generated, discussed, and reshaped as knowledge is shared among team members. Goals that initially reflect a single disciplinary focus evolve into broader objectives that integrate multiple perspectives. Similar to multidisciplinary teams, previous working relationships may exist within the team, contributing to the establishment of shared norms and trust. This trust can help guide the team through both the Development stage and subsequent stages and processes.

In the Conceptualization stage, interdisciplinary teams may undergo multiple iterations of research plan development to capture the nuances of each contributing discipline. Consequently, strategy formulation may take longer than in multidisciplinary teams, as a greater number of ideas and perspectives must be integrated prior to conducting the research. During the Implementation stage, coordination among disciplines is typically more intensive than in multidisciplinary teams, as engaging in the research is more interdependent. The integrated approach requires members to contribute their disciplinary expertise while ensuring that their work remains aligned with the team’s collective goals and objectives. Work may still be performed in parallel; however, team members often rely on output from earlier tasks to inform their own contributions. This interdependence requires the team to monitor task progress more closely than multidisciplinary teams, which can typically operate with greater independence and without requiring input from other members.

Finally, during the Translation stage, interdisciplinary teams emphasize the knowledge gained from their combined approach and consider how the findings can be applied or extended to new avenues of inquiry related to the completed performance cycle. The frameworks and outputs developed at this stage represent the integration of multiple disciplinary perspectives, resulting in a cohesive structure that advances both science and contributing disciplines. However, the translational efforts of interdisciplinary teams do not reach the same threshold as those of transdisciplinary teams, as the application of findings typically remains within disciplinary boundaries rather than leading to entirely novel, cross-disciplinary models. Nevertheless, these efforts play a critical role in guiding future research and may lay the groundwork for future transdisciplinary collaboration. As interdisciplinary teams move through the stages, there is a heavy reliance on the transition process to continually evaluate their alignment with the goals. A large emphasis is also placed during the Implementation phase on the action processes that occur to help support the interdependent nature of the work.

## 9 Conclusion

Our framework provides new theoretical contributions by integrating both organizational and team science research to illustrate where the team process and four-phase frameworks are in alignment and how gaps can be addressed through the establishment of a new, integrated model. Our framework allows for unification in language as researchers continue to study the science of science teams, as well as providing new avenues of empirical research. By using a well-known process model from Marks et al. (2001) and a well-referenced four-phase model of science teaming (Hall et al., 2012), we hope to bridge the gap between team research and the science of teaming. In mapping the two frameworks, we create a more nuanced and holistic approach for scientific teams and the challenges they might experience over time, providing theoretical support for how these challenges have been overcome previously. One constraint of our model is that it has yet to be empirically tested and applied to diverse sets of science teams. We provide propositions to create pathways for empirical testing of our framework, to not only validate it, but to begin developing practices that science teams can engage with to promote improved performance. Additionally, while we account for some contextual factors that influence how science teams work, broader environmental components such as the systems these teams work in and funding need to be further explored.

We acknowledge that the team’s size and the environment in which it works affect the stages and team processes for science teams. In addition, we extend our framework to additional types of science teams, including unidisciplinary, multidisciplinary, and interdisciplinary teams. Thus, mapping these different team types adds illustrations for the implications of team processes and the salience of each phase as it applies to their team composition. Ultimately, our theory-driven integration of team processes and four-phase framework will benefit both researchers wanting to study scientific teams through team processes and help team members understand how individuals coming from different disciplines can work together as a collective to generate greater insights than one discipline could alone.

## Data Availability

The original contributions presented in the study are included in the article/supplementary material, further inquiries can be directed to the corresponding author.

## References

[ref1] Abiew G. E. Okyere-Kwakye E. Ellis F. Y. A. (2022). Examining the effect of functional diversity on organizational team innovation. Int. J. Innov. Sci. 14, 193–212. doi: 10.1108/IJIS-02-2021-0027

[ref2] Bandura A. (1997). Self-efficacy: The exercise of control. New York: Freeman.

[ref3] Bandura A. (2006). Guide for constructing self-efficacy scales. In F. Pajares and T. Urdan (Eds.), Self-efficacy beliefs of adolescents. Greenwich, CT: Information Age Publishing, 307–337.

[ref4] Barsade S. G. Coutifaris C. G. Pillemer J. (2018). Emotional contagion in organizational life. Res. Organ. Behav. 38, 137–151. doi: 10.1016/j.riob.2018.11.005

[ref5] Bates G. Gouais A. L. Barnfield A. Callway R. Hasan M. N. Koksal C. . (2023). Balancing autonomy and collaboration in large-scale and disciplinary diverse teams for successful qualitative research. Int J Qual Methods 22, 1–15. doi: 10.1177/16094069221144594

[ref6] Behfar K. J. Peterson R. S. Mannix E. A. Trochim W. M. K. (2008). The critical role of conflict resolution in teams: a close look at the links between conflict type, conflict management strategies, and team outcomes. J. Appl. Psychol. 93, 170–188. doi: 10.1037/0021-9010.93.1.170, 18211143

[ref79] Bennett L. M. Levine-Finley S. Gadlin H. (2013). Collaboration & team science: a field guide. Available at: http://hdl.handle.net/1885/11070.

[ref7] Bell S. T. Fisher D. M. (2012). Does dynamic composition mean the demise of shared team properties and the rise of global team properties? Ind. Organ. Psychol. 5, 39–41. doi: 10.1111/j.1754-9434.2011.01401.x

[ref8] Benda A. N. Kramer W. S. Baak M. E. Feitosa J. (2023). “Understanding trust in virtual work teams” in Handbook of virtual work. eds. Gilson L. L. O’Neil T. Maynard M. T. (Cheltenham and Northampton, MA: Edward Elgar Publishing Ltd), 305–325.

[ref9] Blomqvist K. Cook K. S. (2018). “Swift trust: state-of-the-art and future research directions” In A.-M. I. Nienaber, R. H. Searle, and S. B. Sitkin (Eds.), The Routledge Companion to Trust. Oxfordshire, United Kingdom: Routledge. 1, 29–49. doi: 10.4324/9781315745572-4

[ref10] Börner K. Contractor N. Falk-Krzesinski H. J. Fiore S. M. Hall K. L. Keyton J. . (2010). A multi-level systems perspective for the science of team science. Sci. Transl. Med. 2, 1–9. doi: 10.1126/scitranslmed.3001399, 20844283 PMC3527819

[ref11] Brown S.-A. Sparapani R. Osinski K. Zhang J. Blessing J. Cheng F. . (2023). Team principles for successful interdisciplinary research teams. Am. Heart J. Plus Cardiol. Res. Pract. 32:100306. doi: 10.1016/j.ahjo.2023.100306, 38510201 PMC10946054

[ref12] Chen Y. Presslee A. Yang S. (2025). The effect of functional diversity on team creativity: behavioral and fNIRS evidence. Manag. Sci. 71, 8007–8026. doi: 10.1287/mnsc.2023.02157

[ref13] Colquitt J. A. Scott B. A. LePine J. A. (2007). Trust, trustworthiness, and trust propensity: a meta-analytic test of their unique relationships with risk taking and job performance. J. Appl. Psychol. 92, 909–927. doi: 10.1037/0021-9010.92.4.909, 17638454

[ref14] Crisp C. B. Jarvenpaa S. L. (2013). Swift trust in global virtual teams. J. Pers. Psychol. 12, 45–56. doi: 10.1027/1866-5888/a000075

[ref15] Cummings J. N. Kiesler S. (2007). Coordination costs and project outcomes in multi-university collaborations. Res. Policy 36, 1620–1634. doi: 10.1016/j.respol.2007.09.001

[ref16] DeChurch L. A. Marks M. A. (2001). Maximizing the benefits of task conflict: the role of conflict management. Int. J. Confl. Manag. 12, 4–22. doi: 10.1108/eb022847

[ref17] Dietl J. E. Derksen C. Keller F. M. Lippke S. (2023). Interdisciplinary and interprofessional communication intervention: how psychological safety fosters communication and increases patient safety. Front. Psychol. 14, 1–14. doi: 10.3389/fpsyg.2023.1164288, 37397302 PMC10310961

[ref18] Edmondson A. (1999). Psychological safety and learning behavior in work teams. Admin. Sci. Q. 44, 350–383. doi: 10.2307/2666999

[ref19] Edmondson A. C. Roloff K. S. (2008). “Overcoming barriers to collaboration: psychological safety and learning in diverse teams” In E. Salas, G. F. Goodwin, and C. S. Burke (Eds.), Team effectiveness in complex organizations (New York: Routledge), 217–242.

[ref20] Elms A. K. Gill H. Gonzalez-Morales M. G. (2023). Confidence is key: collective efficacy, team processes, and team effectiveness. Small Group Res. 54, 191–218. doi: 10.1177/10464964221104218

[ref21] Fawcett J. (2013). Thoughts about multidisciplinary, interdisciplinary, and transdisciplinary research. Nurs. Sci. Q. 26, 376–379. doi: 10.1177/0894318413500408, 24085679

[ref22] Feitosa J. Grossman R. Kramer W. S. Salas E. (2020). Measuring team trust: a critical and meta-analytical review. J. Organ. Behav. 41, 479–501. doi: 10.1002/job.2436

[ref23] Fiore S. M. (2008). Interdisciplinarity as teamwork: how the science of teams can inform team science. Small Group Res. 39, 251–277. doi: 10.1177/1046496408317797

[ref24] Fiore S. M. Salas E. Cannon-Bowers J. A. (2001). “Group dynamics and shared mental model development” in How people evaluate others in organizations. ed. London M. (New York: Lawrence Erlbaum Associates), 309–336.

[ref25] Fiore S. M. Salas E. Cannon-Bowers J. A. (2013). “Group dynamics and shared mental model development” in Edited By Manuel London. How people evaluate others in organizations (New York: Psychology Press), 309–336.

[ref26] Gabelica C. Bossche P. V. den Segers M. Gijselaers W. (2012). Feedback, a powerful lever in teams: a review Educ. Res. Rev. 7 123–144 doi: 10.1016/j.edurev.2011.11.003

[ref27] Hall K. L. Vogel A. L. Huang G. C. Serrano K. J. Rice E. L. Tsakraklides S. P. . (2018). The science of team science: a review of the empirical evidence and research gaps on collaboration in science. Am. Psychol. 73, 532–548. doi: 10.1037/amp0000319, 29792466

[ref28] Hall K. L. Vogel A. L. Stipelman B. A. Stokols D. Morgan G. Gehlert S. (2012). A four-phase model of transdisciplinary team-based research: goals, team processes, and strategies. Transl. Behav. Med. 2, 415–430. doi: 10.1007/s13142-012-0167-y, 23483588 PMC3589144

[ref29] Hamilton K. Mancuso V. Mohammed S. Tesler R. McNeese M. (2017). Skilled and unaware: the interactive effects of team cognition, team metacognition, and task confidence on team performance. J. Cogn. Eng. Decis. Mak. 11, 382–395. doi: 10.1177/1555343417731429

[ref30] Hoegl M. Gemuenden H. G. (2001). Teamwork quality and the success of innovative projects: a theoretical concept and empirical evidence. Organ. Sci. 12, 435–449. doi: 10.1287/orsc.12.4.435.10635

[ref32] Ilgen D. R. Hollenbeck J. R. Johnson M. Jundt D. (2005). Teams in organizations: from input–process–output models to IMOI models. Annu. Rev. Psychol. 56, 517–543. doi: 10.1146/annurev.psych.56.091103.070250, 15709945

[ref31] Jacobs J. P. Wernovsky G. Cooper D. S. Karl T. R. (2015). Principles of shared decision-making within teams. Cardiology in the Young, 25, 1631–1636. doi: 10.1017/s1047951115000311 26282115

[ref33] Jehn K. A. (1995). A multimethod examination of the benefits and detriments of intragroup conflict. Admin. Sci. Q. 40, 256–282. doi: 10.2307/2393638

[ref34] Jiang J. Y. Zhang X. Tjosvold D. (2013). Emotion regulation as a boundary condition of the relationship between team conflict and performance: a multi-level examination. J. Organ. Behav. 34, 714–734. doi: 10.1002/job.1834

[ref35] Kirkman B. L. Mathieu J. E. (2005). The dimensions and antecedents of team virtuality. J. Manage. 31, 700–718. doi: 10.1177/0149206305279113

[ref36] Kozlowski S. W. J. (2015). Advancing research on team process dynamics: theoretical, methodological, and measurement considerations. Organ. Psychol. Rev. 5, 270–299. doi: 10.1177/2041386614533586

[ref37] Kozlowski S. W. Ilgen D. R. (2006). Enhancing the effectiveness of work groups and teams. Psychol. Sci. Public Interest 7, 77–124. doi: 10.1111/j.1529-1006.2006.00030.x, 26158912

[ref38] LePine J. A. Piccolo R. F. Jackson C. L. Mathieu J. E. Saul J. R. (2008). A meta-analysis of teamwork processes: tests of a multidimensional model and relationships with team effectiveness criteria. Pers. Psychol. 61, 273–307. doi: 10.1111/j.1744-6570.2008.00114.x

[ref39] Lewis K. Herndon B. (2011). Transactive memory systems: current issues and future research directions. Organ. Sci. 22, 1254–1265. doi: 10.1287/orsc.1110.0647

[ref40] Love H. B. Fosdick B. K. Cross J. E. Suter M. Egan D. Tofany E. . (2022). Towards understanding the characteristics of successful and unsuccessful collaborations: a case-based team science study. Humanit. Soc. Sci. Commun. 9, 1–11. doi: 10.1057/s41599-022-01388-x

[ref41] Lynn G. Kalay F. (2015). The effect of vision and role clarity on team performance. J. Bus. Econ. Finance 4, 473–499. doi: 10.17261/Pressacademia.2015313067

[ref42] Marks M. A. Mathieu J. E. Zaccaro S. J. (2001). A temporally based framework and taxonomy of team processes. Acad. Manag. Rev. 26:356. doi: 10.2307/259182

[ref43] Marks M. A. Panzer F. J. (2004). The influence of team monitoring on team processes and performance. Hum. Perform. 17, 25–41. doi: 10.1207/S15327043HUP1701_2

[ref44] Marlow S. L. Lacerenza C. N. Salas E. (2017). Communication in virtual teams: a conceptual framework and research agenda. Hum. Resour. Manag. Rev. 27, 575–589. doi: 10.1016/j.hrmr.2016.12.005

[ref45] Martinez Tyson D. Dahlberg B. Higashi R. T. (2025). Bridging two worlds: challenges and opportunities for anthropologists working in team science. Pract. Anthropol. 47, 244–253. doi: 10.1080/08884552.2025.2551588

[ref46] Mathieu J. E. Hollenbeck J. R. van Knippenberg D. Ilgen D. R. (2017a). A century of work teams in the Journal of Applied Psychology. Journal of Applied Psychology, 102, 452–467. doi: 10.1037/apl0000128, 28150984

[ref47] Mathieu J. E. Hollenbeck J. R. van Knippenberg D. Ilgen D. R. (2017b). Team effectiveness 1997–2017: a review of recent advancements and a glimpse into the future. Annu. Rev. Organ. Psych. Organ. Behav. 4, 207–236.

[ref48] Mathieu J. Maynard M. T. Rapp T. Gilson L. (2008). Team effectiveness 1997-2007: a review of recent advancements and a glimpse into the future. J. Manage. 34, 410–476. doi: 10.1177/0149206308316061

[ref49] Mathieu J. E. Rapp T. L. (2009). Laying the foundation for successful team performance trajectories: the roles of team charters and performance strategies. J. Appl. Psychol. 94, 90–103. doi: 10.1037/a0013257, 19186898

[ref50] McLarnon M. J. W. O’Neill T. A. Taras V. Law D. Donia M. B. L. Steel P. (2019). Global virtual team communication, coordination, and performance across three peer feedback strategies. Can. J. Behav. Sci. / Rev. Can. Sci. Comport. 51, 207–218. doi: 10.1037/cbs0000135

[ref51] Mesmer-Magnus J. R. DeChurch L. A. (2009). Information sharing and team performance: a meta-analysis. J. Appl. Psychol. 94, 535–546. doi: 10.1037/a0013773, 19271807

[ref53] Mors M. L. Waguespack D. M. (2021). Fast success and slow failure: the process speed of dispersed research teams. Res. Policy 50:104222. doi: 10.1016/j.respol.2021.104222

[ref54] Natale J. E. Boehmer J. Blumberg D. A. Dimitriades C. Hirose S. Kair L. R. . (2020). Interprofessional/interdisciplinary teamwork during the early COVID-19 pandemic: experience from a children’s hospital within an academic health center. J. Interprof. Care 34, 682–686. doi: 10.1080/13561820.2020.1791809, 32674638

[ref55] National Academies of Sciences, Engineering, and Medicine (2025). The science and practice of team science. Washington DC: The National Academies Press. 40554653

[ref56] Poliseli L. Leite C. M. P. (2021). “Developing transdisciplinary practices” in Global epistemologies and philosophies of science. eds. Ludwig D. Koskinen I. Mncube Z. Poliseli L. Reyes Galindo L. (Milton Park: Routledge), 77–91.

[ref57] Porter C. O. L. H. Hollenbeck J. R. Ilgen D. R. Ellis A. P. J. West B. J. Moon H. (2003). Backing up behaviors in teams: the role of personality and legitimacy of need. J. Appl. Psychol. 88, 391–403. doi: 10.1037/0021-9010.88.3.391, 12814289

[ref58] Rapp T. L. (2025). Team charters: benefits and guidelines for development. Organ. Dyn. 54:101174. doi: 10.1016/j.orgdyn.2025.101174

[ref59] Salas E. Dickinson T. L. Converse S. A. Tannenbaum S. I. (1992). “Toward an understanding of team performance and training” in Teams: Their training and performance. eds. Swezey R. W. Salas E. (Westport, CT: Ablex Publishing), 3–29.

[ref60] Salas E. Reyes D. L. McDaniel S. H. (2018). The science of teamwork: Progress, reflections, and the road ahead. Am. Psychol. 73, 593–600. doi: 10.1037/amp0000334, 29792470

[ref61] Salazar M. R. Lant T. K. Fiore S. M. Salas E. (2012). Facilitating innovation in diverse science teams through integrative capacity. Small Group Res. 43, 527–558. doi: 10.1177/1046496412453622

[ref62] Schwarzer R. Jerusalem M. (1995). “Generalized self-efficacy scale” in Measures in health psychology: A user’s portfolio. Causal and control beliefs. eds. Weinman J. Wright S. Johnston M., Windsor UK: NFER-NELSON. 35–37.

[ref63] Shrum W. Chompalov I. Genuth J. (2001). Trust, conflict and performance in scientific collaborations. Social Stud. Sci. 31, 681–730. doi: 10.1177/030631201031005002

[ref64] Shukla A. K. (2024). Team science: building, nurturing, and expanding research collaborations. Trends Biochem. Sci. 49, 379–381. doi: 10.1016/j.tibs.2023.10.010, 37953092

[ref65] Simon B. (1992). The perception of ingroup and outgroup homogeneity: reintroducing the intergroup context. Eur. Rev. Soc. Psychol. 3, 1–30. doi: 10.1080/14792779243000005

[ref66] Sixsmith J. Fang M. L. Grigorovich A. Wada M. Kontos P. (2020). “Working together as a transdisciplinary team” in A. Sixsmith, J. Sixsmith, A. Mihailidis, M. L. Fang Eds., Knowledge, innovation, and impact: A guide for the engaged health researcher: A guide for the engaged health researcher (Cham: Springer International Publishing), 69–76.

[ref67] Specht A. Crowston K. (2022). Interdisciplinary collaboration from diverse science teams can produce significant outcomes. PLoS One 17:e0278043. doi: 10.1371/journal.pone.0278043, 36445918 PMC9707800

[ref69] Stokols D. Hall K. L. Taylor B. K. Moser R. P. (2008). The science of team science. Am. J. Prev. Med. 35, S77–S89. doi: 10.1016/j.amepre.2008.05.002 18619407

[ref68] Stokols D. Hall K. L. Vogel A. L. (2012). Transdisciplinary public health: Definitions, core characteristics, and strategies for success. American Journal of Preventive Medicine, 42, S77–S89.

[ref70] Tannenbaum S. I. Mathieu J. E. Salas E. Cohen D. (2012). On teams: unifying themes and the way ahead. Ind. Organ. Psychol. 5, 56–61. doi: 10.1111/j.1754-9434.2011.01406.x

[ref71] Tekleab A. G. Quigley N. R. Tesluk P. E. (2009). A longitudinal study of team conflict, conflict management, cohesion, and team effectiveness. Group Organ. Manag. 34, 170–205. doi: 10.1177/1059601108331218

[ref72] Thompson J. L. (2009). Building collective communication competence in interdisciplinary research teams. J. Appl. Commun. Res. 37, 278–297. doi: 10.1080/00909880903025911

[ref73] Tuckman B. W. (1965). Developmental sequence in small groups. Psychol. Bull. 63, 384–399. 14314073 10.1037/h0022100

[ref74] Tzabbar D. Baburaj Y. (2019). Optimizing the effectiveness of geographically dispersed research & development teams. Organ. Dyn. 48, 1–6. doi: 10.1016/J.ORGDYN.2018.09.006

[ref52] Weingart L. R. Cronin M. A. Houser C. J. S. Cagan J. Vogel C. (2000). Functional diversity and conflict in cross-functional product development teams: Considering representational gaps and task characteristics. In L. L. Neider and C. A. Schriesheim (Eds.), Understanding teams. Bingley, UK: Emerald Publishing Limited. 89–110.

[ref75] Wong-Parodi G. Strauss B. H. (2014). Team science for science communication. Proc. Natl. Acad. Sci. USA 111, 13658–13663. doi: 10.1073/pnas.1320021111, 25225381 PMC4183174

[ref76] Yuan Z. Yin J. Sun J. (2025). The paradox of team conflict revisited: an updated meta-analysis of the team conflict–team performance relationships. J. Appl. Psychol. 111, 195–224. doi: 10.1037/apl0001315, 40875336

[ref77] Zeiss A. M. Steffen A. M. (1998). “Interdisciplinary health care teams in geriatrics: an international model” in Comprehensive clinical psychology. eds. Bellack A. S. Hersen M. (Oxford, England: Pergamon), 551–570.

[ref78] Zhang W. Zhang Q. (2014). Multi-stage evaluation and selection in the formation process of complex creative solution. Qual. Quant. 48, 2375–2404. doi: 10.1007/s11135-013-9896-3

